# Novel jack-in-the-box effector of the barley powdery mildew pathogen?

**DOI:** 10.1093/jxb/ery192

**Published:** 2018-06-27

**Authors:** Björn Sabelleck, Ralph Panstruga

**Affiliations:** RWTH Aachen University, Institute for Biology I, Unit of Plant Molecular Cell Biology, Worringerweg, Aachen, Germany

**Keywords:** Barley, *Blumeria graminis* f.sp. *hordei*, effectors, HvMAGAP1, HvRACB, pathogenicity, peptide, powdery mildew, retrotransposon, ROP GTPase

## Abstract

This article comments on:

Nottensteiner M, Zechmann B, McCollum C, Hückelhoven R. 2018. A barley powdery mildew fungus non-autonomous retrotransposon encodes a peptide that supports penetration success on barley. Journal of Experimental Botany **69,** 3745–3758.

**Phytopathogens deploy secreted effector proteins that interfere with different host cellular pathways to suppress plant immunity and redirect nutrient flow. Conventional effectors are small polypeptides with an N-terminal secretion signal, no transmembrane domain and often lacking any protein domain annotation. In their current work,** Nottensteiner *et al.* (2018) **provide experimental evidence for the existence of an unconventional, seemingly retrotransposon-derived effector of the barley powdery mildew fungus. This protein (ROPIP1) interacts with the barley susceptibility factor RACB, possibly leading to local destabilization of cortical microtubular network architecture, thereby supporting fungal host cell entry.**

Powdery mildews are filamentous phytopathogenic fungi that belong to the group of ascomycetes and thrive epiphytically on their plant hosts. They pursue an obligate biotrophic lifestyle, strictly depending on living host tissue for growth and propagation (Glawe, 2008). During infection, these fungi penetrate the cell wall of plant epidermal cells but leave the underlying plasma membrane intact. Inside host cells they form their feeding structure, the haustorium, which is covered by the so-called extrahaustorial membrane – a derivative of the plant plasma membrane (Box 1). Haustoria are probably the sites of fungal nutrient uptake and effector protein delivery, and their establishment is a strict prerequisite for subsequent hyphal growth and sporulation. The intimate association of powdery mildews with their plant hosts at the haustorial interface and their biotrophic lifestyle require an exquisite level of defence suppression, which is considered to be achieved largely via dedicated effector proteins (Hückelhoven and Panstruga, 2011).

**Box 1. Model illustrating the potential mode of action of ROPIP1**

When the barley powdery mildew fungus *Blumeria graminis* f.sp. *hordei* (*Bgh)* successfully establishes its feeding structure (haustorium, grey flask-shaped structure) by penetrating the cell wall (dark green) of a host cell, the underlying plasma membrane (extrahaustorial membrane, red line), which is connected via the neck band (dark grey circles) to the regular host plasma membrane (light green), remains intact and the attacked plant cell stays alive. ROPIP1 (red pentagon) is encoded by a particular class of retrotransposons, so-called *Eg-R1* SINEs (see Box 2), which occur in thousands of copies in the *Bgh* genome. Some of these copies acquired an N-terminal signal peptide by random in-frame insertions in the genome. Following its translation in the fungal pathogen, ROPIP1 is secreted and translocates into host cells via an unknown pathway. There it interacts with the susceptibility factor RACB (brown oval), a small monomeric Rho of plant (ROP) GTPase. RACB in turn interacts with the microtubule-associated ROP GTPase-activating protein (ROP-GAP) MAGAP1 (blue rectangle), which recruits the complex of ROPIP1 and RACB to cortical microtubules (purple lines). This association causes destabilization of the microtubular network via an uncharacterized mechanism. During the plant–­powdery mildew interaction, the latter event might be spatially confined to the actual contact sites of host cells and fungal infection structures.

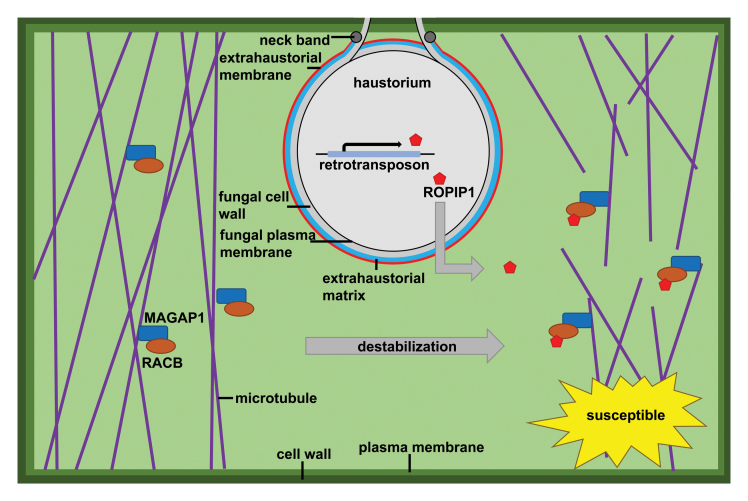

Effectors are small proteins that are secreted by phytopathogens to suppress plant immunity and to promote host colonization (Dou and Zhou, 2012). They are often defined as proteins with an N-terminal secretion signal, no transmembrane domain and no recognizable functional protein domain(s). This group of proteins can be further subdivided, according to their destination in the host, into apoplastic and cytoplasmic effectors. Apoplastic effectors are typically cysteine-rich, likely to withstand the harsh oxidative conditions in the extracellular space and/or to resist plant proteases. Cytoplasmic effectors are delivered into or taken up by host cells via yet poorly resolved mechanisms (Lo Presti and Kahmann, 2017).

*Blumeria graminis* f.sp. *hordei* (*Bgh*) and *B. graminis* f.sp. *tritici* (*Bgt*) are the powdery mildew fungi that colonize the cereals barley and wheat, respectively. Their genomes encode sets of several hundred conventional effector proteins called ‘Candidates for Secreted Effector Proteins’ (CSEPs) and/or ‘Blumeria Effector Candidates’ (BECs) (Pedersen *et al.*, 2012; Wicker *et al.*, 2013; Bindschedler *et al.*, 2016; Frantzeskakis *et al.*, 2018). A subgroup of these CSEPs classify based on their predicted distant structural similarities to ribonucleases and their high expression levels inside haustoria as ‘RNAse-Like Proteins associated with Haustoria’ (RALPHs). This set of effectors might derive from a common ancestral ribonuclease (Spanu, 2017).

Plants evolved the ability to detect some pathogen effectors to trigger a boosted immune response (effector-triggered immunity, ETI). In this case a typically cytoplasmic sensor protein (R protein) recognizes a particular effector variant (then termed avirulence protein, AVR) from the pathogen (Jones and Dangl, 2006). Barley ‘Mildew Locus A’ (MLA) proteins are such R proteins, which belong to the class of intracellular nucleotide-binding domain and leucine-rich repeat proteins (NLRs). For MLA13 and MLA1, two allelic MLA versions, the corresponding *Bgh* AVRs, were identified as classical CSEPs [AVR_a13_ (CSEP0372) and AVR_a1_ (CSEP0008) (Lu *et al.*, 2016)]. However, it is still unknown whether the interaction between R protein and effector is direct or indirect in these instances. So far, there are only a few documented examples of direct interactions between *Bgh* effector candidates and host plant proteins (Zhang *et al.*, 2012; Schmidt *et al.*, 2014; Ahmed *et al.*, 2015; Pennington *et al.*, 2016).

## Transposable elements – a source of new effectors?

The genome of the barley powdery mildew pathogen *Bgh* is about four-times larger than the median genome size of other ascomycetes, but its gene number is smaller than in most other filamentous fungi. Around 75% of the genomic DNA consists of transposable elements (TEs), which are evenly distributed throughout the genome (Spanu *et al.*, 2010; Amselem *et al.*, 2015*a* ; Frantzeskakis *et al.*, 2018). This unusually high amount probably reflects the absence of the repeat-induced point mutation (RIP) mechanism, which usually controls the spread of TEs, and this could account for the huge genome size (Spanu *et al.*, 2010). TEs generally fall into class I (retrotransposons) or class II (DNA transposons). In powdery mildews, class I retrotransposons dominate (Spanu *et al.*, 2010; Amselem *et al.*, 2015*a* ). Retrotransposons are subdivided into those with long terminal repeats (LTRs) as well as long and short interspersed nuclear elements (LINEs and SINEs, respectively) that lack LTRs and are therefore collectively termed non-LTR retrotransposons (Box 2). While LTR retrotransposons and LINEs are autonomous, SINEs are non-autonomous, i.e. they cannot transpose on their own (Finnegan, 2012).

**Box 2. Classification and modular structure of retrotransposons**

Class I transposons (retrotransposons) are divided in two main groups, LTR retrotransposons (from a few hundred base pairs up to 25 kb in size) and non-LTR retrotransposons. The non-LTR retrotransposons can be further subdivided into LINEs and SINEs. LTR (dark blue) refers to ‘long terminal repeats’, which are a few hundred base pairs long and flank the central part of the TE as direct repeats. This kind of retrotransposon harbours two to three ORFs between the LTRs. The *gag* (*group-specific antigen*) gene (green) product forms a virus-like particle, while *pol* (orange) encodes a polyprotein that undergoes cleavage and comprises a combination of an integrase, a reverse transcriptase and an RNase H domain. Some LTR retrotransposons have also an *env* gene (grey), which codes for a (typically defective) viral envelope protein.

LINEs are typically 6–8 kb in size and composed of two ORFs, where ORF1 (light blue) encodes an RNA-binding protein that is necessary for the LINE transposition intermediate and ORF2 (purple) codes for a nuclease and a reverse transcriptase. In some cases, an RNase H domain is also included in ORF2. At the beginning of a LINE retrotransposon is a promoter that regulates transcription of the ORFs, and at the 3′ end is a polyA signal.

SINEs (~300 bp long) have no coding regions and harbour a Box A and a Box B, which show similarity with an internal RNA polymerase III promoter. These retrotransposons rely on LINE-encoded reverse trancriptases for their proliferation. They show sequence similarity at the 5′ ends to tRNAs and at the 3′ ends to the distal part of LINEs. Note that the various types of retrotransposons are not drawn to scale.

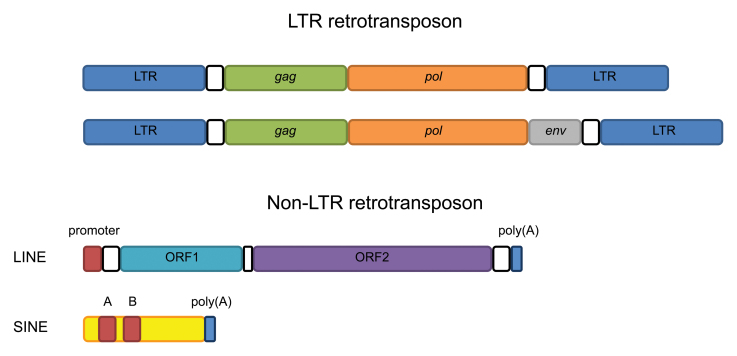

Until now it was thought that TEs primarily generate an enormous playground for the birth and death of conventional effector variants, e.g. via non-homologous (illegitimate) recombination events (Raffaele and Kamoun, 2012; Gladieux *et al.*, 2014; Grandaubert *et al.*, 2014; Fouche *et al.*, 2018). However, in their present study, Nottensteiner *et al.* (2018) show that a peptide, which apparently derives from a SINE-related TE termed *Eg-R1* (Wei *et al.*, 1996), acts as an unconventional effector of *Bgh*. This peptide was originally found in a yeast two-hybrid (Y2H) screen for interactors of RACB, a small monomeric Rho of plant (ROP)-type GTPase that serves as a susceptibility factor in barley (Schultheiss *et al.*, 2002). Using RACB as a bait, the peptide was retrieved multiple times from a prey cDNA library that was established from barley leaves heavily infected with *Bgh*. The authors demonstrate that the peptide can interact with RACB in a targeted Y2H test and also *in planta*, as shown by ‘bimolecular fluorescence complementation’ (BiFC). In both assays a dominant-negative form of RACB, characterized by a single amino acid substitution (T20N), served as a specificity control (Kudla and Bock, 2016). Because of the interaction and its small size (74 amino acids), they named this peptide ‘ROP Interactive Peptide 1’ (ROPIP1). The respective open reading frame (ORF) is associated with the 5′ end of *Eg-R1*, which probably occurs in several thousand copies in the *Bgh* genome. At least some of these copies appear to have in-frame 5′ extensions that encode predicted canonical secretion signals.

The group validated pathogen-induced *ROPIP1* transcript accumulation using qRT-PCR analysis and detected infection-dependent protein accumulation in immunoblot analysis using a polyclonal antiserum raised against a synthetic epitope peptide located in the C-terminus of ROPIP1. Notably, the authors also performed *in situ* immunodetection of ROPIP1 via transmission electron microscopy-based immunogold labelling. This technique revealed the presence of ROPIP1-associated gold label in fungal infection structures (appressoria and haustoria), but also within the host cell wall, local cell wall appositions (papillae) and the host cytoplasm, suggesting secretion of ROPIP1 from the fungal pathogen into host cells. Results from transient overexpression and gene silencing experiments in single transformed barley epidermal cells hint at a role of ROPIP1 in fungal virulence since overexpression increased and silencing conversely decreased fungal penetration success. The specificity of the latter effect was confirmed with a silencing-resistant ‘RNAi rescue’ construct. The authors went on to demonstrate the fate of ROPIP1 in host cells by studying the subcellular localization of a fluorophore-tagged version. When co-expressed with the known RACB interactor MAGAP1, ROPIP1 localizes to cortical microtubules. RACB promotes the recruitment of ROPIP1 via MAGAP1 to microtubules, which leads to a dramatic alteration in microtubular network architecture, characterized by a substantial increase of cells with fragmented microtubules. The authors claim that this destabilization of the cortical microtubular network, which in the authentic interaction might be spatially confined to the site of attempted host cell entry, may aid fungal virulence (Box 1).

## ROPIP1 – a jack-in-the-TE-box?

ROPIP1 is not the first effector nominee allegedly originating from a powdery mildew retrotransposon. In an earlier study, candidates for the avirulence proteins AVR_a10_ and AVR_k1_ were reported to derive from a particular class of LINE-1 retrotransposons present in the *Bgh* genome (Ridout *et al.*, 2006). The elements encoding the respective family of proteins – termed EKAs (Effectors homologous to AVR_k1_ and AVR_a10_) – were similarly to *Eg-R1* found to occur in >1000 copies in the *Bgh* genome (Sacristan *et al.*, 2009; Amselem *et al.*, 2015*b* ). However, the existence of EKA-related proteins was only indirectly shown by the occurrence of respective peptide fragments upon proteomic analysis of samples from infected plant epidermis and fungal hyphae (Amselem *et al.*, 2015*b* ), and plant cellular targets and/or a potential mode of effector mechanism remained elusive. Moreover, the absence of prototypical N-terminal secretion signals in this protein family raised concerns regarding their potential for transfer into host cells. By contrast, the presence of prototypical secretion sequences in a subset of the ROPIP1 copies, the immunological detection of ROPIP1 in fungal and host cells, the identification of candidate host targets as well as a suggested cellular activity altogether make up a plausible scenario (Box 1). Accordingly, some *Bgh Eg-R1* versions might have been neofunctionalized to support fungal virulence.

It remains, however, puzzling that despite the many putative *ROPIP1* copies in the *Bgh* genome, which show apparent length polymorphism regarding the encoded proteins, a discrete signal is seen in immunoblot analysis. This raises the possibility that only one or a few of the many potential ROPIP1 variants is/are produced by fungal cells. Although some copies seem to harbour potentially functional secretion signals, another formal possibility is that ROPIP1 is secreted via a non-conventional secretion pathway (Wang *et al.*, 2018) as discussed by the authors. Given its non-canonical origin, future research activities are needed to corroborate (i) the actual existence of the ROPIP1 peptide (e.g. by protein mass spectrometry of immunoprecipitated protein), (ii) its transfer into host cells (via conventional or unconventional secretion pathways?) and (iii) its *in planta* interaction with the RACB/MAGAP1/microtubular network complex (e.g. via fluorescence resonance energy transfer (FRET) studies or by co-immunoprecipitation analysis). But even with these caveats in mind, the work by Nottensteiner and co-workers provides interesting food for thought.
